# Supplementary figures and images for: If you build it, they will come: rapid colonization by dragonflies in a new effluent-dependent river reach
Source: PeerJ. 2020 Sep 16;8:e9856. doi: 10.7717/peerj.9856 (PMC7501785; doi:10.7717/peerj.9856)

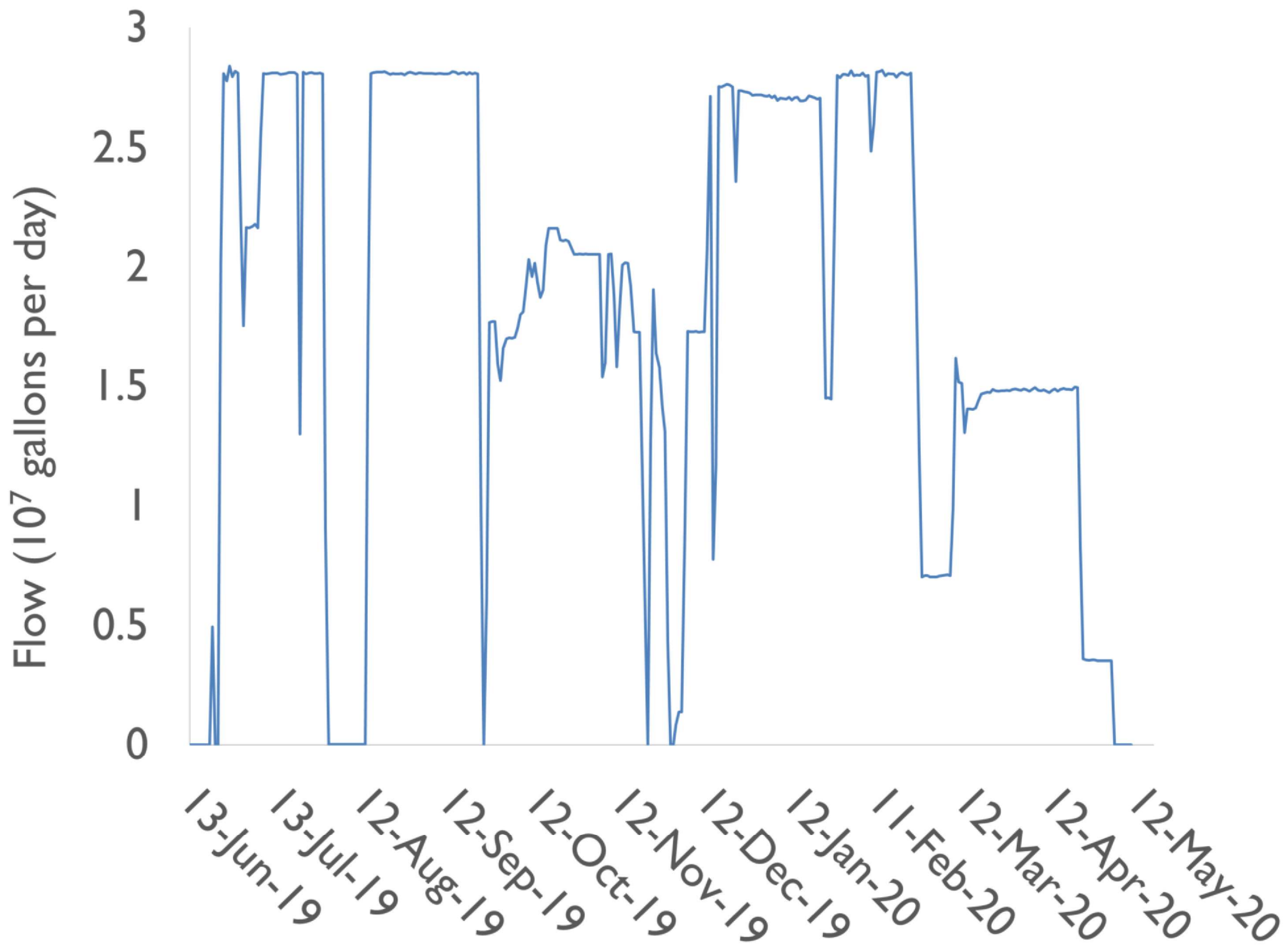

Supplement: Supplemental Information 1 — Effluent volume release data for the Heritage project from 24 June 2019 to 29 April 2020. When flow dropped below 1.5 million gallons day−1 (5.7 million liters day−1) for 24 h, then the Cushing study site would begin to dry up. When flow dropped below 0.5 gallons day−1 (1.9 million liters day−1) for more than 24 h, the Starr Pass site would begin to dry up. Data courtesy of Tucson Water (City of Tucson, AZ). [file peerj-08-9856-s001.pdf]
